# Genome-wide analysis of the mouse lung transcriptome reveals novel molecular gene interaction networks and cell-specific expression signatures

**DOI:** 10.1186/1465-9921-12-61

**Published:** 2011-05-02

**Authors:** Rudi Alberts, Lu Lu, Robert W Williams, Klaus Schughart

**Affiliations:** 1 Department of Infection Genetics, Helmholtz Centre for Infection Research & University of Veterinary Medicine Hannover, Inhoffenstr. 7, D-38124 Braunschweig, Germany; 2 Department of Anatomy and Neurobiology, University of Tennessee Health Science Center, Memphis, Tennessee, USA; 3 Jiangsu Key Laboratory of Neuroregeneration, Nantong University, Nantong, China

## Abstract

**Background:**

The lung is critical in surveillance and initial defense against pathogens. In humans, as in mice, individual genetic differences strongly modulate pulmonary responses to infectious agents, severity of lung disease, and potential allergic reactions. In a first step towards understanding genetic predisposition and pulmonary molecular networks that underlie individual differences in disease vulnerability, we performed a global analysis of normative lung gene expression levels in inbred mouse strains and a large family of BXD strains that are widely used for systems genetics. Our goal is to provide a key community resource on the genetics of the normative lung transcriptome that can serve as a foundation for experimental analysis and allow predicting genetic predisposition and response to pathogens, allergens, and xenobiotics.

****Methods**:**

Steady-state polyA+ mRNA levels were assayed across a diverse and fully genotyped panel of 57 isogenic strains using the Affymetrix M430 2.0 array. Correlations of expression levels between genes were determined. Global expression QTL (eQTL) analysis and network covariance analysis was performed using tools and resources in GeneNetwork http://www.genenetwork.org.

****Results**:**

Expression values were highly variable across strains and in many cases exhibited a high heri-tability factor. Several genes which showed a restricted expression to lung tissue were identified. Using correlations between gene expression values across all strains, we defined and extended memberships of several important molecular networks in the lung. Furthermore, we were able to extract signatures of immune cell subpopulations and characterize co-variation and shared genetic modulation. Known QTL regions for respiratory infection susceptibility were investigated and several *cis*-eQTL genes were identified. Numerous *cis*- and *trans-*regulated *trans*cripts and chromosomal intervals with strong regulatory activity were mapped. The *Cyp1a1 *P450 *trans*cript had a strong *trans*-acting eQTL (LOD 11.8) on Chr 12 at 36 ± 1 Mb. This interval contains the *trans*cription factor *Ahr *that has a critical mis-sense allele in the *DBA/2J *haplotype and evidently modulates transcriptional activation by AhR.

****Conclusions**:**

Large-scale gene expression analyses in genetic reference populations revealed lung-specific and immune-cell gene expression profiles and suggested specific gene regulatory interactions.

## Background

The lung is the first line of defense against many pathogens and inhaled xenobiotics and is therefore a key part of the immune system. Host defense is strongly influenced by genetic differences and several studies have shown that the genetic background and sequence difference among humans and other host species modulate susceptibility and resistance to infectious diseases, allergens, and xenobiotics. Systems genetics is a modern extension of complex trait analysis that jointly analyzes and integrates large sets of genotypes and phenotypes to explain and predict variation in outcome measures and disease severity (for review see [1,2]). A typical systems genetics study relies on extensive single nucleotide polymorphism (SNP) data sets, matched data on RNA expression in key cells, tissues, or organs and a core set of key dependent measures such as disease susceptibility [3]. These data are collected across a panel or population of genetically diverse individuals or strains. This group of individuals represents a natural genetic perturbation, with well defined genotype and haplotype differences comprising the "treatment." The independent measurements in this case can consist either of the genotype or of crucial intervening variables such as the expression of genes and proteins.

In this study, we exploited a very well characterized panel of inbred strains of mice (a mouse genetic reference panel) that consists of two parts--a small set of standard inbred strains and a larger family of BXD type recombinant inbred strains. The genome of each BXD strain represents a mixture of the C57BL/6J and DBA/2J parental background and is homozygous at almost every genomic location. The genomic make-up of each BXD line has been determined by extensive mapping with molecular markers. After performing microarray expression analysis for each of the BXD mice, the expression level of each gene can be used as a quantitative trait (*e.g. *[4-6]). By comparing these expression values for all BXD mice with their molecular markers data along the genome, genomic expression quantitative trait loci (eQTL) can be identified that are likely to regulate the expression of one or several genes [2,5,7-12]. When an eQTL is located at the same genomic position as the gene itself (within a 10Mb interval of the gene) it is considered as a *cis*-eQTL. In this case, variations in the promoter sequence or in elements that determine the stability of the mRNA of the gene are the most likely causes for the observed differences in expression levels. If the eQTL is at a distant location from the regulated gene, the eQTL is referred to as a *trans*-eQTL.

Here, we performed a global gene expression analysis from the lungs of 47 BXD and eight widely used inbred strains. The aim of our study was to reveal genes and gene networks in mouse lung in steady state condition. We found that many genes had high variation in expression and that often this variation was highly heritable. This allowed us to identify many *cis- *and *trans-*eQTLs. In addition, we used the correlation structure in the data to obtain expression signatures for specific cell types within the lung.

## Methods

### Mouse strains and sample preparation

C57BL/6J, BALB/cByJ, FVB/NJ, and WSB/EiJ, as well as B6D2F1 and D2B6F1 lines were obtained from the University of Tennessee Health Science Center (UTHSC). DBA/2J, 129X1/SvJ, LP/J and SJL/J were obtained from The Jackson Laboratory. Mice from 38 BXD recombinant inbred strains were obtained from UTHSC and mice from nine BXD strains were obtained from The Jackson Laboratory. All animals were housed at UTHSC before sacrifice. Mice were killed by cervical dislocation and whole lungs including blood were removed and placed in RNAlater. Total RNA was extracted from the lungs using RNA STAT-60 (Tel-Test Inc.). RNA from two to five animals per strain were pooled and used for gene expression analysis. Animals used in this study were between 49 and 93 days of age. All inbred strains were profiled for both sexes, and for a given BXD strain either males or females were used. Mice were maintained under specific pathogen free conditions. All protocols involving mice were approved by the UTHSC Animal Care and Use Committee.

### Microarray analysis

Gene expression profiling was performed using Affymetrix GeneChip Mouse Genome 430 2.0 Arrays at UTHSC. Samples were amplified according to the recommended protocols by the manufacturer (Affymetrix, Santa Clara, CA, USA). In all cases, 4-5 μg of each biotinylated cRNA preparation was fragmented and included in a hybridization cocktail containing four biotinylated hybridization controls (BioB, BioC, BioD, and Cre), as recommended by the manufacturer. Samples were hybridized for 16 hours. After hybridization, GeneChips were washed, stained with SAPE, and read using an Affymetrix GeneChip fluidic station and scanner.

### Data preprocessing and analysis

Data analysis was performed using the GeneNetwork web service [13], a large resource with phenotypes and mRNA expression data for several genetic reference populations and multiple organisms. The expression data were preprocessed like all other datasets in GeneNetwork: adding an offset of 1 unit to each signal intensity value to ensure that the logarithm of all values were positive, computing the log_2 _value, performing a quantile normalization of the log_2 _values for the total set of arrays using the same initial steps used by the RMA transform [14], computing the Z scores for each cell value, multiplying all Z scores by 2 and adding 8 to the value of all Z scores. The advantage of this variant of a Z transformation is that all values are positive and that 1 unit represents approximately a 2-fold difference in expression as determined using the spike-in control probe sets (see [8] for details). For correlation analyses we used Pearson's correlation unless otherwise stated. Heritability was determined using ANOVA with one factor mouse strain, and by dividing the mean between-mouse-strain variance by the sum of the mean between-mouse strain variance plus the mean within-strain variance.

### QTL Mapping and expression analyses

All probe sets were mapped using standard interval mapping methods at 1 cM intervals (~2 Mb) along all autosomes and the X chromosome. This procedure generates estimates of linkage between variation in transcript expression levels and chromosomal location. The entire set of values can be used to construct a set of QTL maps for all chromosomes (except Chr Y and the mitochondrial genome) in which position is plotted on the x-axis and the strength of linkage--the likelihood ratio statistic (LRS) or log of the odds ratio (LOD)-is plotted on the y-axis. An LRS of 18 or higher is significant at a genome-wide p value of < 0.5. To compute LRS values we exploited the computationally efficient Haley-Knott regression equations [15] and a set of 3796 SNPs and microsatellite markers that we and others have genotyped over the past decade [16,17]. In order to rapidly map all 45,101 probe sets we used our customized QTL Reaper code http://qtlreaper.sourceforge.net/. QTL Reaper performs up to a million permutations of an expression trait to calculate the genome-wide empirical *p *value and the LRS scores associated with each interval or marker. The peak linkage value and position was databased in GeneNetwork and users can rapidly retrieve and view these mapping results for any probe set. Any of the QTL maps can also be rapidly regenerated using the same Haley-Knott methods, again using functions imbedded in GeneNetwork. GeneNetwork also enable a search for epistatic interactions (pair scanning function) and composite interval mapping with control for a single marker.

### Data quality control

We used two simple but effective methods to confirm correct sample identification of all data entered into GeneNetwork. Expression of the *Xist *transcript (probe set 1427262_at) was used to validate the sex of the sample. *Xist *is involved in the inactivation of one X chromosome in females [18] and is only expressed at high levels in females. Other genes that show strong sex-specific expression are *Eif2s3y*, *Jarid1d *and *Ddx3y*. In addition, we investigated several genes that exhibit a strongly bimodal Mendelian expression pattern, meaning that one parental allele exhibits a high expression level whereas the other allele exhibits a low expression and only the F1 hybrids are intermediate. The expression level of such transcripts is directly correlated with the genotype at this locus and they can collectively be used to confirm sample genotype and hence strain. For example, expression of the *Rpgrip1 *transcript (probe set 1421144_at) has a distinctly bimodal distribution, intermediate values for F1 animals, and is associated with a LOD score peak of 50 that corresponds precisely to the location of the cognate gene on Chr 14 at 52.5 Mb.

## Results

### Variation in gene expression

The Affymetrix M430 2.0 array that we used includes 45,101 probe sets and provides consensus estimates of expression for the vast majority of all protein coding genes. Table 1 gives an overview of the range of variation across strains in each of the probe sets used. Strikingly, more than 2,000 genes showed a range of expression that was larger than four-fold different between the strain with the lowest and the highest expression. Among the genes with the most extreme range in expression levels were *Krt4 *(keratin 4), *Krt13 *(keratin 13) and *Krtdap *(keratinocyte differentiation associated protein). Another gene with highly variable expression was *Cftr *(cystic fibrosis transmembrane conductance regulator homolog). This important lung disease-causing gene showed a four-fold variation in expression levels between strains. Several other genes with high variation were sex-specifically expressed genes, like *Xist *(inactive X specific transcripts), *Ddx3y *(DEAD (Asp-Glu-Ala-Asp) box polypeptide 3, Y-linked) and *Serpina1b *(serine (or cysteine) preptidase inhibitor, clade A, member 1B).

**Table 1:** Variation in gene expression for 45,101 probe sets.

Fold change range	Log_2 _range	No. of genes
1-2	0-1	30,392
2-4	1-2	11,965
4-8	2-3	1,980
8-16	3-4	498
16-32	4-5	132
32-64	5-6	60
64-inf	6-inf	42

Fold changes between the lowest and highest expressed mouse stains per probe sets were cal-culated and divided in seven bins. The corresponding range on log_2 _scale and the amount of genes in each range are given.

### Heritability of variation in gene expression

To investigate to which extent the variation in expression was due to genetic effects, we calculated the heritability for each of the genes, which is the fraction of variation in expression caused by genetics. The heritability values ranged from as high as 0.96 (most of the variance was associated with between-strain differences) until as low as 0.01. Genes with the largest heritability were *Cdk17/Pctk2 *(cyclin-dependent kinase 17, probe set 1446130_at), *Gm1337 *(predicted gene 1337, 1443287_at) and *Pdxdc1/KIAA0251 *(pyridoxal-dependent decarboxylase domain containing 1, 1452705_at), all having a value above 0.99. High heritability values indicate that it is likely to successfully map QTLs that influence gene expression values.

### Lung-specific genes

**Figure 1:**
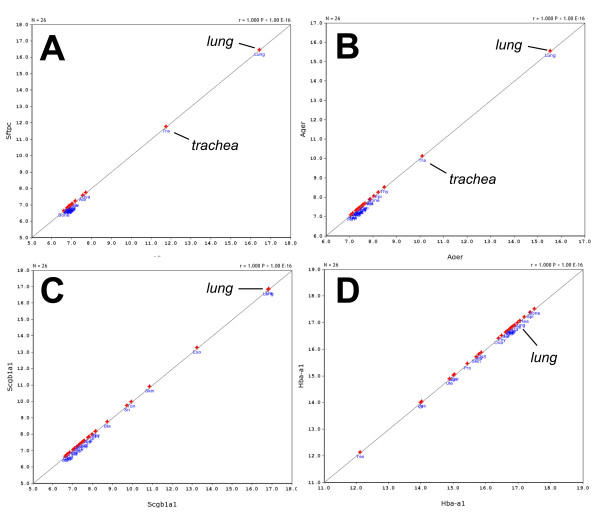
**Tissue distribution in 25 other tissues of some of the most highly expressed genes in the lung**. The expression levels for (A) *Stpc*, (B) *Ager*, (C) *Scgb1a1 *and (D) *Hba-a1 *in different tissues are shown. Please note that in this representation the gene is correlated with itself to illustrate only its issue distribution.

The large dataset in GeneNetwork and its built-in features allowed us to compare the gene ex-pression patterns in the lung with data from 25 other tissues. First, we identified the most highly expressed genes in lung (Table 2 lists the 15 highest expression signals). Two of these genes were highly restricted to the lung and trachea: *Sftpc *(surfactant associated protein C) and *Ager *(advanced glycosylation end product-specific receptor) (Figure 1A, B) whereas *Scgb1a1 *(secretoglobin, family 1A, member 1 (uteroglobin)) was highly expressed in lung but also showed expression in some other tissues (Figure 1C). On the other hand, *Hba-a1 *(hemoglobin alpha, adult chain 1) was expressed at high levels in most tissues (Figure 1D). We then used *Stfpc *in a tissue correlation analysis to identify other genes that may not be as highly expressed but still be restricted to lung tissue. The first 70 probe sets found were then analyzed as above for lung-specific expression, and 15 genes were identified (Table 3). A comparison to the expression patterns described in the BioGPS database [19] confirmed that the majority was only expressed in lung, most of them at high level. Two genes were not restricted to the lung according to BioGPS, and five genes were also found at lower levels in one other tissue (Table 3).

**Table 2:** List of 15 probe sets with highest expression signals in the lung.

Probe set	Symbol	Description	Location (Chr, Mb)	Mean Expr	Tissue-specific expression
1428361_x_at	*Hba-a1*	hemoglobin alpha, adult chain 1	Chr11: 32.184441	15,10	MT
1418639_at	*Sftpc*	surfactant associated protein C	Chr14: 70.920826	14,92	LS
1452543_a_at	*Scgb1a1*	secretoglobin, family 1A, member 1 (uteroglobin)	Chr19: 9.158206	14,78	LHOT
1417184_s_at	*Hbb-b2*	hemoglobin, beta adult minor chain	Chr7: 110.976103	14,74	MT
1441958_s_at	*Ager*	advanced glycosylation end product-specific receptor	Chr17: 34.737745	14,69	LS
AFFX-b-ActinMur/M12481 _3_at	*Actb*	actin beta, cytoplasmic	Chr5: 143.665528	14,67	MT
1452757_s_at	*Hba-a1*	hemoglobin alpha, adult chain 1	Chr11: 32.196742	14,66	MT
1416642_a_at	*Tpt1*	tumor protein, translationally-controlled 1	Chr14: 76.246098	14,62	MT
1418509_at	*Cbr2*	carbonyl reductase 2	Chr11: 120.628111	14,62	LHOT
1436996_x_at	*Lzp-s*	P lysozyme structural and lysozyme	Chr10: 116.724902	14,62	ND
1416624_a_at	*Uba52*	ubiquitin A-52 residue ribosomal protein fusion product 1	Chr8: 73.032191	14,58	MT
1427021_s_at	*Fth1*	ferritin heavy chain 1	Chr19: 10.057382	14,57	ND
AFFX-MURINE_B2_at	*B2*	AFFX-MURINE_B2_at short interspersed nuclear element (SINE) class of repeat (probes target Chr 1 and Chr 2 most heavily)	N/A	14,52	ND
1415906_at	*Tmsb4x*	thymosin, beta 4, X chromosome	ChrX: 163.645132	14,51	MT
1449436_s_at	*Ubb*	ubiquitin B	Chr11: 62.366564	14,50	MT

Mean Expr: mean expression in lung for BXD strains. LS: lung specific expression, LHOT: highly expressed in lung but also in other tissues, MT: expressed in many tissues or mainly in non-lung tissues, ND: no data for other tissues than lung available.

**Table 3:** List of genes with lung-restricted expression found by tissue correlation analysis with *Sftpc*.

Probe set	Symbol	Description	Location (Chr, Mb)	Mean Expr	BioGPS expression
1418639_at	*Sftpc*	surfactant associated protein C	Chr14: 70.920826	14,92	high in lung, low in nucleus accumbens
1437028_at	*Sftpb*	surfactant associated protein B (nonciliated bronchiolar and alveolar type 2 cell signature)	Chr6: 72.260763	13,68	high in lung only
1422334_a_at	*Sftpa1*	surfactant associated protein A1	Chr14: 41.946994	14,24	high in lung only
1422346_at	*Nkx2-1 (Titf1)*	thyroid *trans*cription factor 1	Chr12: 57.634187	8,07	lung only
1417057_a_at	*Lamp3*	lysosomal-associated membrane protein 3	Chr16: 19.653875	12,09	high in lung, low in ES cells and some cell lines
1421404_at	*Cxcl15*	chemokine (C-X-C motif) ligand 15	Chr5: 91.230349	13,87	high in lung only
1441958_s_at	*Ager*	advanced glycosylation end product-specific receptor	Chr17: 34.737745	14,69	high in lung only
1436787_x_at	*Sec14l3*	SEC14-like protein 3	Chr11: 3.978573	13,21	only data for human available - not lung specific
1425218_a_at	*Scgb3a2*	secretoglobin, family 3A, member 2	Chr18: 43.924081	14,17	high in lung only
1449428_at	*Cldn18*	claudin 18	Chr9: 99.591247	12,70	highest in lung, lower in stomach
1449525_at	*Fmo3*	flavin containing monooxygenase 3	Chr1: 164.884088	10,90	high in lung, maybe weak in some other tissues
1425814_a_at	*Calcrl*	calcitonin receptor-like	Chr2: 84.170818	12,91	high in lung, weak in macrophages
1421373_at	*Cox4i2*	cytochrome c oxidase subunit IV isoform 2	Chr2: 152.582819	9,24	not specific for lung
1419699_at	*Scgb3a1*	secretoglobin, family 3A, member 1	Chr11: 49.477871	13,68	high in lung only
1451604_a_at	*Acvrl1*	activin A receptor, type II-like 1	Chr15: 100.968668	11,86	high in lung only
1420347_at	*Plunc*	palate, lung, and nasal epithelium carcinoma associated	Chr2: 153.973359	13,42	high in lung, low in heart

Mean Expr: mean expression in lung for BXD strains. BioGPS: evaluation of expression pattern as described in BioGPS.

### Identification of gene networks using correlations

**Figure 2:**
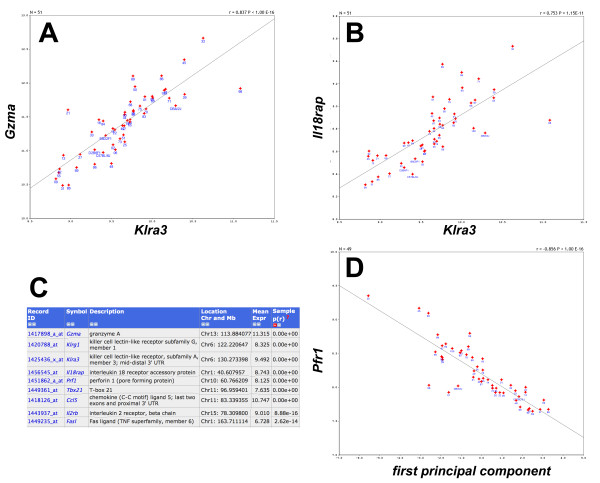
**Expression signals for strongly correlated genes in the lung**. Numbers indicate BXD strains, and the parental C57BL/6J and DBA/2J strains as well as F1 individuals are presented. Expression signals of (A) *Klra3 *and *Gzma *(p < 10^-16^) and (B) *Klra3 *versus *Il18rap *(p < 10^-11^) were strongly correlated. (C) List of nine genes highly correlated with the first principal component of the expression of *Gzma*, *Klrg1*, *Klra3 *and *Il18rap*. (D) Strong correlation between the first principal component and *Prf1 *(p < 10^-16^). X- and Y-axis of the plots show the names of genes used for the analysis.

The large data set for expression values for ~39,000 transcripts in 57 mouse strains allowed us to calculate correlations between any pair of genes. A Spearman rank correlation analysis identified 12,985 pairs of genes with a correlation value above 0.8, and 604 pairs showed a correlation value of 0.9 or higher. For example, the expression of *Klra3 *(killer cell lectin-like receptor subfamilily A, member 3) was strongly correlated with the expression of *Gzma *(granzyme A) (Figure 2A). *Klra3 *also appeared to be strongly correlated with *Il18rap *(interleukin 18 receptor accessory protein, Figure 2B). We then calculated the first principal component of the *Klra3*, *Gzma*, *Il18rap *and *Klrg1 *(killer cell lectin-like receptor subfamily G, member 1) genes and used it to determine the correlations with all other genes in the lung data set. In this way, we could identify a network of nine genes exhibiting a correlation of >= 0.8 with this principal component (Figure 2C). One of the newly identified genes was *Prf1 *(perforin 1) which was correlated with a p-value of < 10^-16 ^with the principal component (Figure 2D). If genes exhibit a strong correlation of their expression values, one may hypothesize that they are involved in the same biological process or pathway, or they may be expressed in the same cell type.

**Figure 3:**
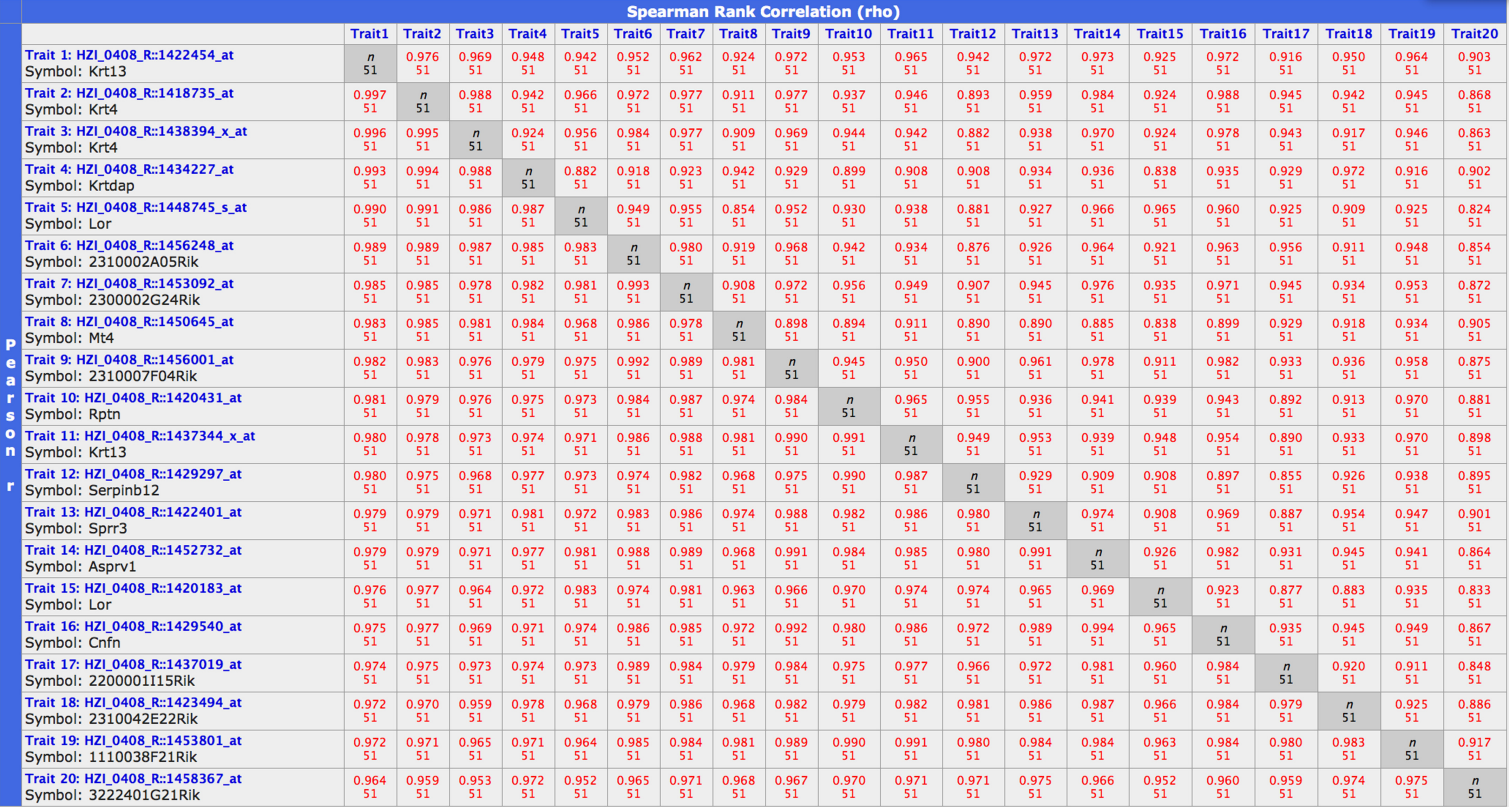
**The cytokeratin network**. Pearson correlations (listed below the diagonal) showed very high correlations between all pairs of the 20 selected genes. Spearman Rank correlations are shown above the diagonal.

In a similar way, we identified another gene network of 20 genes that exhibited very high correlations of their expression levels across all mouse strains. All possible pairs of genes in this network showed a correlation above 0.95 (Figure 3). The network contained two keratin genes, *Krt4 *(keratin 4) and *Krt13 *(keratin 13) and genes involved in cytoskeleton functions, again pointing to a possible interaction of these genes in the same pathway or biological process. Further gene networks found by correlation studies were related to B and T cells (see below).

### Correlation analysis identified gene expression signatures for T and B cells

**Figure 4:**
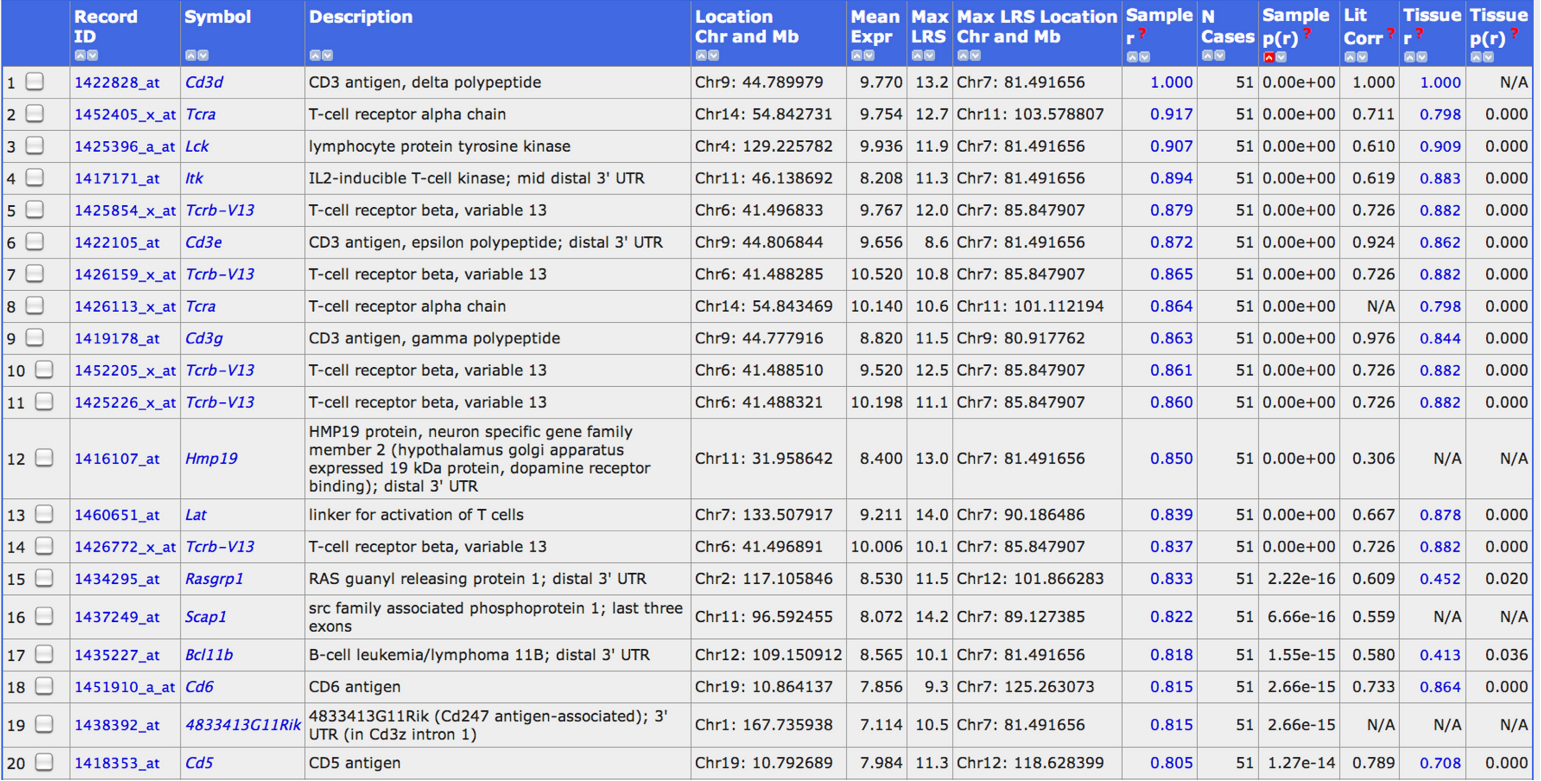
**Gene signatures for T-cells**. List of the strongest correlates for *Cd3d *(probe set 1422828_at), all correlated at p < 10^-13^.

**Figure 5:**
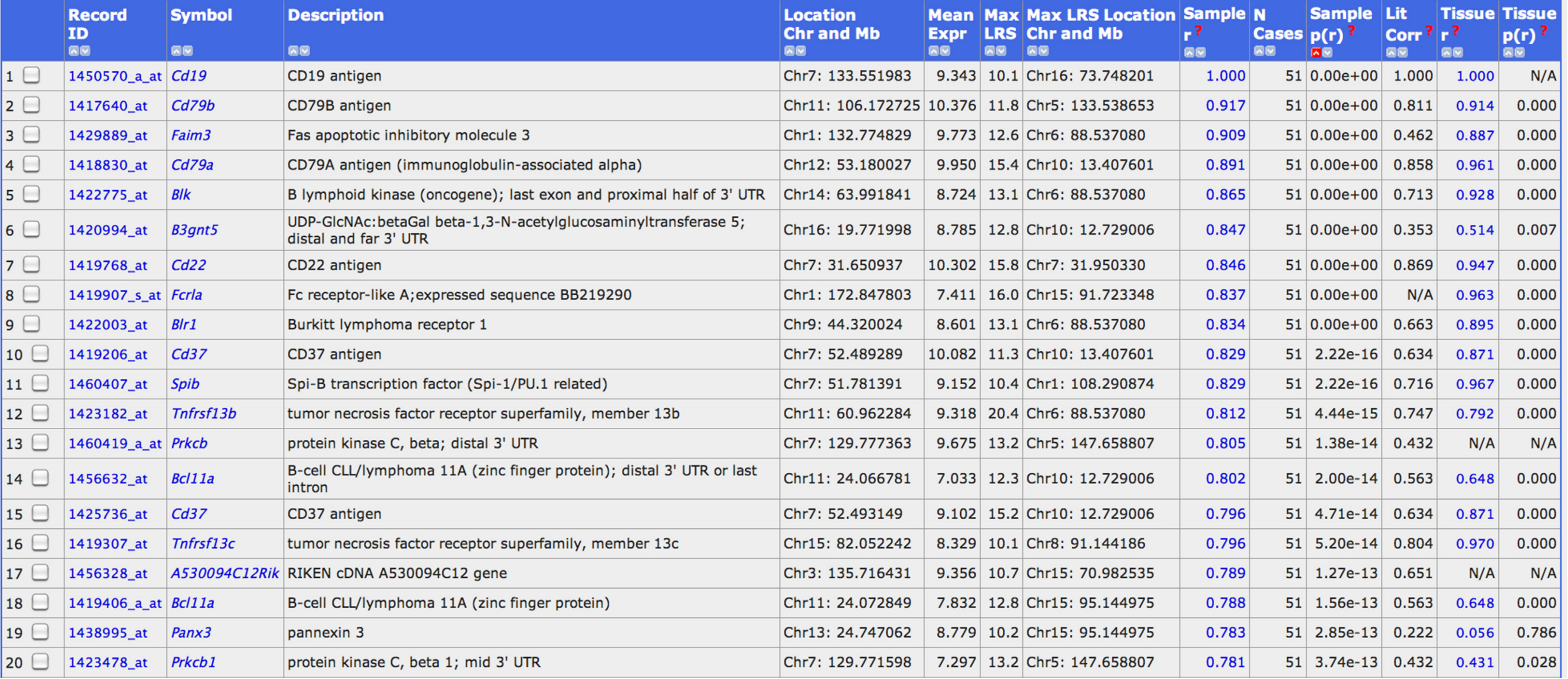
**Gene signatures for B-cells**. List of the strongest correlates for *Cd19 *(probe set 1450570_at), all correlated at p < 10^-12^.

The hemoglobin genes *Hba-a1 *(hemoglobin alpha, adult chain 1) and *Hbb-b2 *(hemoglobin beta, adult minor chain) were among the top 10 genes with highest expression values in our lung data set. The high levels of hemoglobin transcripts suggested that circulating blood cells, including immune cells, may also be analyzed in our data set. Therefore, we investigated the gene expression networks of known immune cell markers, *e.g*. *Cd3 *genes as specific markers for T cells. We calculated the correlations of *Cd3d *(Cd3 antigen, delta polypeptide) expression levels over all BXD lines with all other genes. This analysis revealed 20 genes with a very highly correlated expression value (p-value below 10^-14^, Figure 4). Most of these genes were known T cell markers or involved in T cell regulation. Eight out of the 12 genes with the strongest correlations were also exclusively expressed in T cells according to the BioGPS database (Wu et al., 2009): *Cd3d, Itk, Tcrb-13V Cd3e, Cd3g, Scap1, Cd6 and Cd5 *(see Figure 4 for full gene names). Similarly, we searched for B cell-specific signatures starting with the B cell marker gene *Cd19 *(CD19 antigen). The probe set "1450570_a_at" detected *Cd19 *mRNA levels and showed a mean expression level of 9.3. We found 14 probe sets with a correlation above 0.80 (p-value < 10^-14^, Figure 5). A comparison with the BioGPS database revealed that eight of them, *Cd19, Cd79b, Faim3, Cd79a, Blk, B3gnt5, Cd22 and Blr1 (*see Figure 5 for full gene names) were also exclusively expressed in B cells. Therefore, these genes can be considered as T and B cell signature genes which may be used to follow the presence and infiltration of T and B cells in the lung under normal and pathological conditions.

### Identification of candidate genes regulating phenotypic traits in the lung

Once a QTL for a phenotypic trait has been found, it will be important to identify the underlying quantitative gene (QTG) which is causing the variation. Searching *cis*-eQTLs in the QTL interval represents one suitable approach [8]. As a prototype for this approach in our lung data set, we examined two traits for which lung phenotypes were studied in the BXD population and which were available in GeneNetwork. Boon et al. [20] described several QTLs for the susceptibility of BXD mice to influenza A infections. We analyzed one significant QTL peak on chromosome 2 and two suggestive peaks on chromosomes 7 and 17. Seven *cis*-eQTL regulated genes were found in the chromosome 2 QTL interval (Table 4), including the *Hc *(hemolytic complement) gene which was shown to contribute to influenza susceptibility [20]. The analysis of the QTL region on chromosome 7 revealed 12 *cis*-regulated genes in the lung, including *Trim12 *(tripartite motif protein 12) and *Trim34 *(tripartite motif protein 34) which were also described as potential candidate QTGs by [20]. In the chromosome 17 QTL region, we found 17 *cis*-eQTL genes, of which *Prkcn *(protein kinase C, nu), *Qpct *(glutaminyl-peptide cyclo*trans*ferase (glutaminyl cyclase) and *Mta3 *(metastasis associated 3) were suggested as potential QTGs by [20]. Another lung-specific phenotype in the GeneNetwork database is "Mycoplasmosis susceptibility, alveolar exudate" (GeneNetwork ID 10692, [21] and Cartner et al. unpublished). This trait showed a significant QTL on chromosome 10, between 105 and 130 Mb. The analysis of our lung expression data revealed 16 *cis*-eQTLs in the genomic interval (Table 5). Three of the *cis*-QTL genes have been associated previously with immune functions and thus represent suitable candidates to regulate this trait: *Chst *(carbohydrate (keratan sulfate Gal-6) sulfotransferase 1) was found to exhibit a critical role in lymphocyte trafficking during chronic inflammation [22]. The transcription factor *Maf *(avian musculoaponeurotic fibrosarcoma (v-maf) AS42 oncogene homolog) was shown to play a role in the transcriptional regulation of cytokine expression and immune cell markers, e.g. [23-29]. *Nrp1 *(neuropilin 1) has been primarily described as neuronal receptor but appears also to play a role in the primary immune response and formation of the immunological synapse [30,31].

**Table 4:** *Cis*-eQTLs identified in QTL inteval on chromosome 2 for influenza susceptibility.

Probe set	Symbol	Description	Location (Chr, Mb)	Mean Expr	Max LRS
1423602_at	*Traf1*	Tnf receptor-associated factor 1	Chr2: 34.798805	9,28	21,1
1419407_at	*Hc*	hemolytic complement	Chr2: 34.838908	12,00	82,7
1441635_at	*Nr6a1*	nuclear receptor subfamily 6, group A, member 1	Chr2: 38.736451	7,51	20,2
1455743_at	*Olfml2a*	olfactomedin-like 2A	Chr2: 38.816929	8,28	63,2
1430379_at	*Zfhx1b*	zinc finger homeobox 1b	Chr2: 44.931019	9,08	82,4
1438516_at	*Rif1*	Rap1 interacting factor 1	Chr2: 51.975068	8,03	38,8
1444530_at	*Neb*	nebulin	Chr2: 51.991339	8,14	86,9

For each gene, only the highest LRS is shown. Mean Expr: mean expression in lung for BXD strains.

**Table 5:** *Cis*-eQTLs identified in QTL on chromosome 8 for Mycoplasmosis susceptibility trait.

Probe set	Symbol	Description	Location (Chr, Mb)	Mean Expr	Max LRS
1435883_at	*AW413431*	expressed sequence AW413431	Chr8: 109.374192	8,27	37
1436986_at	*Sntb2*	syntrophin, basic 2	Chr8: 109.537595	6,94	23,6
1437003_at	*5730419I09Rik*	RIKEN cDNA 5730419I09 gene	Chr8: 109.543026	9,79	24,6
1451052_at	*Cog8*	component of oligomeric golgi complex 8	Chr8: 109.570082	10,35	23,7
1417766_at	*1810044O22Rik*	RIKEN cDNA 1810044O22 gene	Chr8: 109.710789	11,80	38
1429725_at	*Atbf1*	AT motif binding factor 1	Chr8: 111.481987	8,84	70,1
1453393_a_at	*Chst4*	carbohydrate (chondroitin 6/keratan) sulfo*trans*ferase 4	Chr8: 112.553165	7,33	71,9
1427513_at	*Nudt7*	nudix (nucleoside diphosphate linked moiety X)-type motif 7	Chr8: 116.678269	6,95	22,7
1446412_at	*Wwox*	WW domain-containing oxidoreductase	Chr8: 117.339587	7,46	94,7
1444073_at	*Maf*	avian musculoaponeurotic fibrosarcoma (v-maf) AS42 oncogene homolog	Chr8: 118.225461	7,93	121
1449964_a_at	*Mlycd*	malonyl-CoA decarboxylase (test Mendelian in BXDs with high DBA/2J allele)	Chr8: 121.934407	9,63	34,8
1418856_a_at	*Fanca*	Fanconi anemia, complementation group A	Chr8: 125.792224	7,98	78,5
1460109_at	*D8Ertd325e*	DNA segment, Chr 8, ERATO Doi 325, expressed	Chr8: 125.915951	7,60	89,8
1449307_at	*Dbndd1*	dysbindin (dystrobrevin binding protein 1)	Chr8: 126.029666	7,14	24,1
1446982_at	*Pard3*	par-3 (partitioning defective 3) homolog (C. elegans)	Chr8: 130.036847	8,02	87,9
1448944_at	*Nrp1*	neuropilin 1	Chr8: 131.027919	11,95	42,4

For each gene, only the highest LRS is shown. Mean Expr: mean expression in lung for BXD strains.

### Cis- and trans-eQTLs

**Figure 6:**
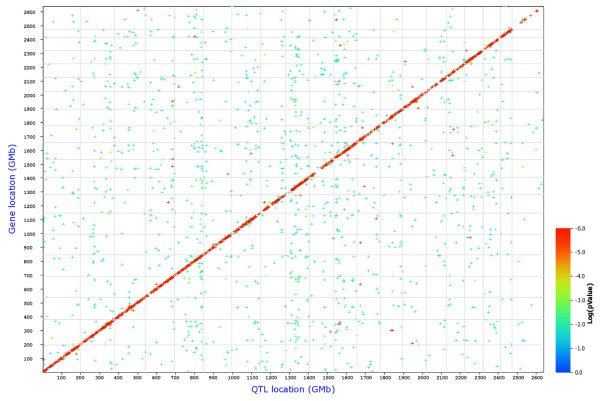
**Genome-wide graph of *cis- *and *trans-*eQTLs**. The positions of the eQTLs are plotted against the locations of the corresponding *trans*cript along the genome. *Cis*-regulated genes are located at the diagonal, all other dots represent *trans*-regulated genes. The significance level of each QTL is indicated by the color.

**Figure 7:**
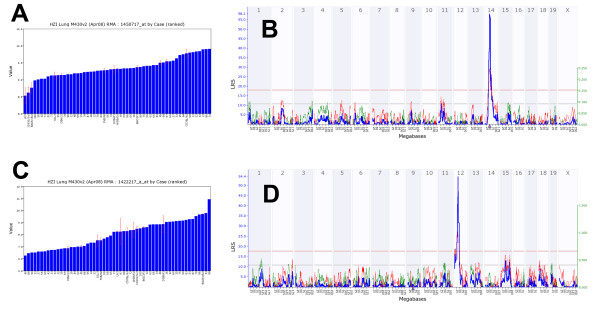
**Examples for variations of expression levels in different BXD and inbred mouse strains and ge-nome-wide analysis of *cis- *and *trans*-eQTLs**. (A) Log_2 _expression levels for *Ang1 *and (B) corresponding *cis*-eQTL signal. The numbers at the top are chromosomes. The blue line represents the significance level of the QTL expressed as LRS score (likelihood ratio statistic). A positive additive coefficient (green line) indicates that DBA/2J alleles increased trait values. A negative additive coefficient (red line) indicates that C57BL/6J alleles increased trait values. The two horizontal lines mark the genome-wide significance levels at p < 0.05 (red line) and p < 0.63 (gray line). *Ang1 *is located on Chr 14 (triangle) and the QTL peak is at the same location as the gene. (C) Log_2 _expression levels for *Cyp1a1 *and (D) corresponding *trans *eQTL peak. *Cyp1a1 *is on chromosome 9 (triangle) and the QTL was found on chromosome 12.

**Figure 8:**
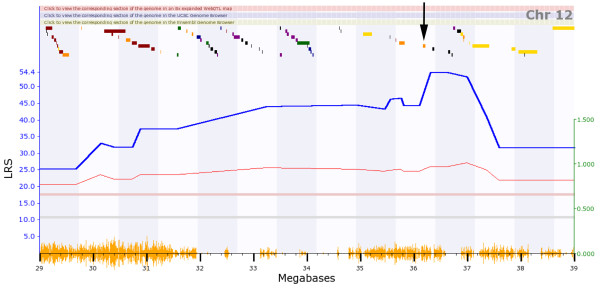
**The *trans*cription factor *Ahr *is located within the *trans*-eQTL region for *Cyp1a1***. The strongest eQTL for *Cyp1a1 *maps to Chromosome 12 and the QTL peaks between 36 and 37 megabases. The gene *Ahr*, indicated by an arrow, is located exactly at the top of the QTL peak.

We then performed a search for eQTLs on a global level, for all probe sets. In this analysis 5,214 *cis- *and 15,485 *trans-*regulated genes were identified at an LRS threshold of 12 (Table 6 and Figure 6). When the LRS threshold was increased to 50, 1,332 *cis*-regulated genes were found, whereas the number of *trans*-regulated genes was reduced to 15. This observation indicates that many of the *trans*-eQTL showed a much lower significance value than the *cis*-eQTL. Next, we present examples for one *cis- *and one *trans-*eQTL. A strong eQTL was detected on chromosome 14, at 52 megabases (Mb; Figure 7B) regulating the expression levels of *Ang *(angiogenin, ribonuclease, RNase A family, 5) (Figure 7A). Since *Ang *is located at the same position as the eQTL (51.7 Mb on chromosome 14) it represents a *cis*-eQTL. Furthermore, a strong eQTL was found on chromosome 12 regulating the expression levels of the *Cyp1a1 *gene (cytochrome P450, family 1, subfamily a, polypeptide 1) (Figure 7C,D). *Cyp1a1 *is located on chromosome 9 and the corresponding eQTL was found on chromosome 12 (*trans*-eQTL). The eQTL significance interval contained nine genes, four of which were expressed in lung at a level above 10. *Ahr *(aryl-hydrocarbon receptor) was one of the four genes and was at the top of the QTL peak (Figure 8). It is the most likely candidate for *Cyp1a1 *regulation. In conclusion, our data set contained a large number of genes whose expression levels are likely to be influenced by allelic variations in the genomes of C57BL/6J and DBA/2J. Therefore, the presence of pairs of regulated genes and their corresponding eQTLs predicts possible regulatory interactions and will allow searching for yet unknown regulatory networks.

**Table 6:** Amount of *cis*- and *trans*-regulated *trans*cripts for different significance thresholds

Threshold (LRS)	No. of *cis *eQTLs	No. of *trans *eQTLs
12	5,214	15,485
16	4,391	3,149
20	3,666	536
30	2,500	48
50	1,332	15

## Discussion

Here, we performed global gene expression profiling in eight inbred mouse strains and a cohort of BXD recombinant inbred strains from whole lung tissues. Our studies identified several lung-specific genes, large variations in gene expression levels, and a strong heritability in many gene expression traits. Correlation analysis of gene expression and genotypes identified potential gene interaction networks, pairs of *trans*- and *cis*-eQTLs, and genes with *cis*-eQTLs that may represent candidate genes involved in susceptibility to respiratory infections. In addition, one specific gene interaction pathway was identified in which *Ahr *regulates the *Cyp1a1 *gene.

Using tissue correlations of gene expression patterns across the BXD strains, we identified 16 genes with a highly restricted expression in the lung of which 14 could be validated by comparison to the BioGPS database [19]. The second most strongly expressed gene in the lung tissues was *Sftpc *which has been shown to play a role in lung development and the prevention of pneumonitis and emphysema [32,33]. Also, *Sftpc *deficiency increases the severity of respiratory syncytial virus-induced pulmonary inflammation [34]. Furthermore, *Scgb1a1 *and *Ager *were amongst the five most strongly expressed genes. *Scgb1a1 *is expressed in lung clara cells and its deficiency results in enhanced susceptibility to environmental agents [35]. *Scgb3a1 *(secretoglobin, family 3A, member 1) and *Scgb3a2 *(secretoglobin, family 3A, member 2) were shown by others to be highly expressed in the lung and lower levels in other organs [36]. *Scgb3a2 *is down-regulated in inflamed airways [37] and plays an important role in lung development [38]. *Sftpb *(surfactant associated protein B (non-ciliated bronchiolar and alveolar type 2 cell signature) is a hydrophobic peptide which enhances the surface properties of pulmonary surfactant and is expressed in non-ciliated bronchiolar and aleveolar type 2 cells [39]. Maintenance of *Sftpb *expression is critical for survival during acute lung injury [40] and reduction of alveolar expression causes surfactant dysfunction and respiratory failure [41]. *Plunc *(palate, lung, and nasal epithelium carcinoma associated) is expressed in the oral, lingual, pharyngeal and respiratory epithelia [42] and members of the *Plunc *gene family are thought to pay a role in the innate immune response [43]. The presence of Plunc protein in the lung decreases the levels of *Mycoplasma pneumoniae *and its levels are reduced in allergic inflammatory conditions [44]. Thus, the lung data set allowed us to find important genes that are expressed primarily in the lung and are important for lung homeostasis and prevention of disease.

It should be noted that our analysis of genes with "restricted expression to the lung" is not ex-clusive; it only refers to the tissues that are represented in GeneNetwork and BioGPS. Also, the analysis performed here should not be considered to be comprehensive. More sophisticated approaches may be employed to identify additional genes which also fulfill the criterion of "lung-restricted" expression.

Furthermore, genes may not be apparent in the lung transcriptome because they are expressed only in a small fraction of cells within the lung. This issue of dilution of expression signals is an important one and we have studied it in several tissues with considerable care (eye, retina, and numerous brain regions) using the same genetic methods and the same array platform. We were consistently able to detect expression of genes that are only expressed in very small cell subpopulations (<0.1%) such as rare amacrine cell subclasses in the retina [8] or very rare oxytocin-expressing neurons (<2000) in whole brain samples. The reason for the increased sensitivity is that with such large sample sizes (~70 lung arrays) the signal-to-noise ratios are much better than standard studies using Affymetrix arrays. These stuides typically use far fewer arrays and do not use genetic methods to "validate" the source of signal.

The strong signal for hemoglobin and lymphocyte-specific genes clearly showed that gene ex-pression patterns of circulating blood cells are readily detectable in the lung transcriptome. This raises the question if an organ should be studied with or without containing blood. The correct answer to this question depends of course on the particular circumstances. However, we feel strongly that a global systems and genetic approach requires the analysis of the entire organ. The expression of genes is not cell-autonomous and depends on cellular micro envi-ronment, physical factors (gas pressure and gradients, etc), pathogen exposure, and many types of interactions. These factors also influence the expression of genes in blood cells. Therefore, we think that it is imperative to look simultaneously at all cells in a function unit: in this case the whole lung plus its containing blood.

In conclusion, the combined analysis of expression levels and correlations in a variety of tissues tissue allowed us to determine genes with restricted or preferential expression in the lung. For several of these genes, an important function in the lung has been described and the same may be assumed for the others. This information will also contribute to a better understanding of the biological function of these genes.

Many phenotypic traits have been studied for the BXD mouse populations and several QTLs were identified which influence diseases or vulnerability in the lung. The detection of *cis *eQTLs in the very same tissue is one method to identify potential candidate genes under the QTL which may causally influence the trait. Here, we investigated two traits in more detail, susceptibility to influenza virus and susceptibility to mycoplasmosis. Several *cis*-eQTLs were found in the corresponding QTL regions and in each case, genes could be identified with a presumed role in the host immune defense (discussed already in the results section). Thus, the study of *cis*-eQTLs in our data set may provide valuable candidates for other quantitative trait genes that influence important lung phenotypes. Furthermore, we found 13 BXD lines with low expression signals for *Krt4*, *Krt13 *and *Krtdap*. *Krt4 *and *Krt13 *have been shown to be responsible for White sponge nevus (WSN), also known as Cannon's disease, which is an autosomal dominant skin condition in humans [45-47]. We propose that the 13 mouse strains have genetic alterations which result in low transcript levels of these genes and they may represent a good model for Cannon's disease. It should be noted, however, that no *cis*-eQTLs found were found for any of the *Krt *genes.

We also identified a set of genes for which the expression levels correlated highly with members of the *Klr *gene family. *Klra3 *and *Klrg1 *are killer cell lectin-like receptors that are exclusively expressed on natural killer cells (NK cells). NK cells form a major component of the innate immune system and kill cells by releasing small cytoplasmic granules of proteins called perforins and granzymes [48]. Both *Gzma *and *Prf1 *were in the gene network that we identified. In addition, correlations can also be used to expand already known gene networks in specific cell populations. When starting with the *Cd3 *T cell marker and calculating correlations with all other transcripts measured, we identified a strongly correlated network of genes, in which most of the genes were known as T cell markers or to be involved in T cell activation or homeostasis. In a similar way, when starting with the *Cd19 *B cell marker, we could identify a strongly correlated network of B cell signature genes. The analysis of these T cell and B cell expression signatures in the Bi-oGPS data base with expression profiles in mouse tissues revealed that indeed >90% of the T and B cell markers were specifically expressed in either T or B cells. Furthermore, most of the T and B cell signature genes represented genes with known function in B and T cell differentiation, activation and homeostasis. For example, the T cell signature included genes encoding subunits of the T cell receptor: *Cd3d *(CD3 antigen, delta po-lypeptide), *Cd3g *(CD3 antigen, gamma polypeptide), *Tcra *(T-cell receptor alpha chain) and *Tcrb-V13 *(T-cell receptor beta, variable 13) and *Lat *(linker for activation of T cells) which are involved in T cell activation. The B cell signature contained components of the B cell antigen receptor complex, *Cd19 *(CD19 antigen) and *Cd79a *(CD79A antigen (immunoglobulin-associated alpha)), as well as *Blk *(B lymphoid kinase) tyrosine kinase which is associated with the receptors. Also, the correlations for both signatures in the spleen expression data set in GeneNetwork could indeed confirm that the signatures were strongly correlated (data not shown). In summary, these studies demonstrate that correlation analyses are able to identify genes which very likely interact in a common network or biological process. The approach used here may thus have a great potential to identify new networks and biological processes in the lung. In addition, starting with a known bona-fide cell-specific gene and then analyzing gene expression values across strains, it is possible to identify a set of highly correlated genes. These gene sets genes can now be used as cell-specific signature genes in complex transcriptome studies, *e.g. *to detect infiltrating immune cells in the lungs after infection.

The genetic mapping of lung expression profiles revealed many *cis- *and *trans-*eQTLs, indicating that many gene expression patterns in lung have a strong genetic component. *Trans*-eQTLs allow the identification of gene-gene regulatory networks. As an example, we found that the transcription factor *Ahr *was present in a *trans*-eQTL region detected for the *Cyp1a1 *gene. *Ahr *is a transcription factor known to induce *Cyp1a1 *transcription levels after ligand binding [49-51]. Six binding sites for the Ahr receptor ligand have been revealed in the 700-basepair DNA domain upstream of *Cyp1a1 *[52]. However, a critical leucine-to-proline substitution in *Ahr *results in a 15 to 20-fold reduction in the binding affinity of the proline variant found in DBA/2J compared to the leucine variant found in C57BL/6J [53]. Indeed, in our data set, expression values for *Cyp1a1*were low for BXD strains carrying the DBA/2J allele at the *Ahr *locus and high for the strains carrying the C57BL/6J allele. Since *Ahr *is not *cis*-regulated in lung, the downstream effects appear to be only caused by changes in Ahr protein binding affinity. Although the interaction between *Cyp1a1 *and *Ahr *as such is not a new finding, it is quite remarkable that the interaction becomes apparent in lungs which were not exposed to an inducing xenobiotic. Furthermore, we do not see this relationship in several other tissues, such as liver. Therefore, our observation suggests that in the lung, which is potentially exposed to many xenobiotics, the Ahr receptor may always be activated at a low level. Alternatively, *Ahr *expression may be stimulated by yet unknown ligands that are also present under normal environmental conditions.

## Conclusions

Here, we showed that whole genome expression analysis of the lungs from a large set of strains of the BXD mouse population can be exploited to identify important gene regulatory networks. We found a large number of expression correlations and QTLs which can be further investigated to better understand molecular interaction networks in the lung. The search for *cis*-eQTLs in genomic intervals that were identified previously as QTLs for infectious diseases revealed several quantitative trait candidate genes. In addition, we demonstrated that the analysis of gene expression correlations, starting with a few cell-specific genes, could identify a larger set of genes which allows detecting the presence of B and T cells within the transcriptome of the whole lung. Such expression signatures will be very important to follow normal and abnormal host responses during infections and other diseases of the lung.

## Competing interests

The authors declare that they have no competing interests.

## Authors' contributions

RA performed the bioinformatics analysis and wrote the manuscript. KS designed the experiments, performed the bioinformatics analysis and wrote the manuscript. LL and RWW prepared the study material and supervised the expression array studies. RW contributed to writing of the manuscript. All authors have read and approved the final manuscript.
