# Genome-wide analysis of the mouse lung transcriptome reveals novel molecular gene interaction networks and cell-specific expression signatures

**DOI:** 10.1186/1465-9921-13-5

**Published:** 2012-01-19

**Authors:** Rudi Alberts, Lu Lu, Robert W Williams, Klaus Schughart

**Affiliations:** 1 Department of Infection Genetics, Helmholtz Centre for Infection Research & University of Veterinary Medicine Hannover, Inhoffenstr. 7, D-38124 Braunschweig, Germany; 2 Department of Anatomy and Neurobiology, University of Tennessee Health Science Center, Memphis, Tennessee, USA; 3 Jiangsu Key Laboratory of Neuroregeneration, Nantong University, Nantong, China

## Text

After publication of our article [1], we became aware of a typographical error in the Methods section, which concerned the statement about the p-value of an LRS of 18 or higher in our QTL analysis. Below is the first part of the corresponding chapter with the corrected p-value (marked in bold). We apologize for any inconvenience.

### QTL Mapping and expression analyses

All probe sets were mapped using standard interval mapping methods at 1 cM intervals (~2 Mb) along all autosomes and the X chromosome. This procedure generates estimates of linkage between variation in transcript expression levels and chromosomal location. The entire set of values can be used to construct a set of QTL maps for all chromosomes (except Chr Y and the mitochondrial genome) in which position is plotted on the x-axis and the strength of linkage--the likelihood ratio statistic (LRS) or log of the odds ratio (LOD)-is plotted on the y-axis. An LRS of 18 or higher is significant at a genome-wide **p-value of < 0.05**.
